# Thoracic Pedicle Screw Placement Utilizing Hands-On Training Session on Three-Dimensional Models

**DOI:** 10.7759/cureus.28544

**Published:** 2022-08-29

**Authors:** Tye Patchana, Ajay Ramnot, Saman Farr, Andrew Ku, Muhammad Ghauri, Andrew Crouch, Dan E Miulli

**Affiliations:** 1 Neurosurgery, Riverside University Health System Medical Center, Moreno Valley, USA; 2 Neurosurgery, Desert Regional Medical Center, Palm Springs, USA; 3 Neurosurgery, California University of Science and Medicine, Colton, USA; 4 Emergency Medicine, Arrowhead Regional Medical Center, Colton, USA; 5 Neurosurgery, Arrowhead Regional Medical Center, Colton, USA

**Keywords:** 3d models, three-dimensional models, medical education, pedicle screw placement, thoracic spine

## Abstract

The utilization of three-dimensional (3D) models has been an important element of medical education. We demonstrate a three-dimensionally-printed (3DP) thoracic spine model for use in the teaching of freehand pedicle screw placement. Neurosurgical residents with varying years of experience practiced screw placement on these models. Residents were timed, and models were evaluated for medial and lateral breaches. Overall, this technical report describes the utility of 3D spine models in the training of thoracic pedicle screw placement. The tactile feedback from the 3D models was designed to represent both cortical and cancellous bones.

## Introduction

Computer-aided design (CAD) complemented by three-dimensional (3D) printing allows the creation of objects with minimal waste and lower material costs [1]. With the expense of traditional cadaveric models of human anatomy, 3D printing may offer a more economical alternative for teaching and training residents. There are abundant sources depicting the optimal placement of pedicle screws within the thoracic spine. Optimal screw placement in the thoracic spine is paramount given the unique anatomy at this location. Because of this, training for freehand placement of thoracic pedicle screws takes on unique importance that has been emphasized in the literature [2]. With the advent of 3D models, its applications have arisen, especially within the medical sector. Previous studies utilizing 3D-printed (3DP) thoracic models for the practice of epidural placement have shown to have positive responses and feedback from trainees [3].

We propose the use of 3D-printed thoracic spine models to assess the feasibility of its utilization in training neurosurgery residents on how to place thoracic pedicle screws. Six 3D-printed thoracic models were used, each with six levels of pedicles available for screw placement. Residents were tabulated into cohorts by post-graduate year (PGY) and received brief instruction, followed by screw placement. These same residents will then receive detailed instructions provided by neurosurgery attendings. Following the attending’s instruction, residents again attempted to place screws. Screw placement was assessed by attending physicians, specifically for medial breaches into the central canal or lateral breaches through the pedicle or vertebral body. Following this exercise, a survey was conducted to assess the utility of using 3D-printed spine models for screw placement and further assess the realistic nature of the models. Data such as the efficiency of screw placement, PGY, and pre-and post-attending instruction efficiency of screw placement were calculated.

## Technical report

**Figure 1:**
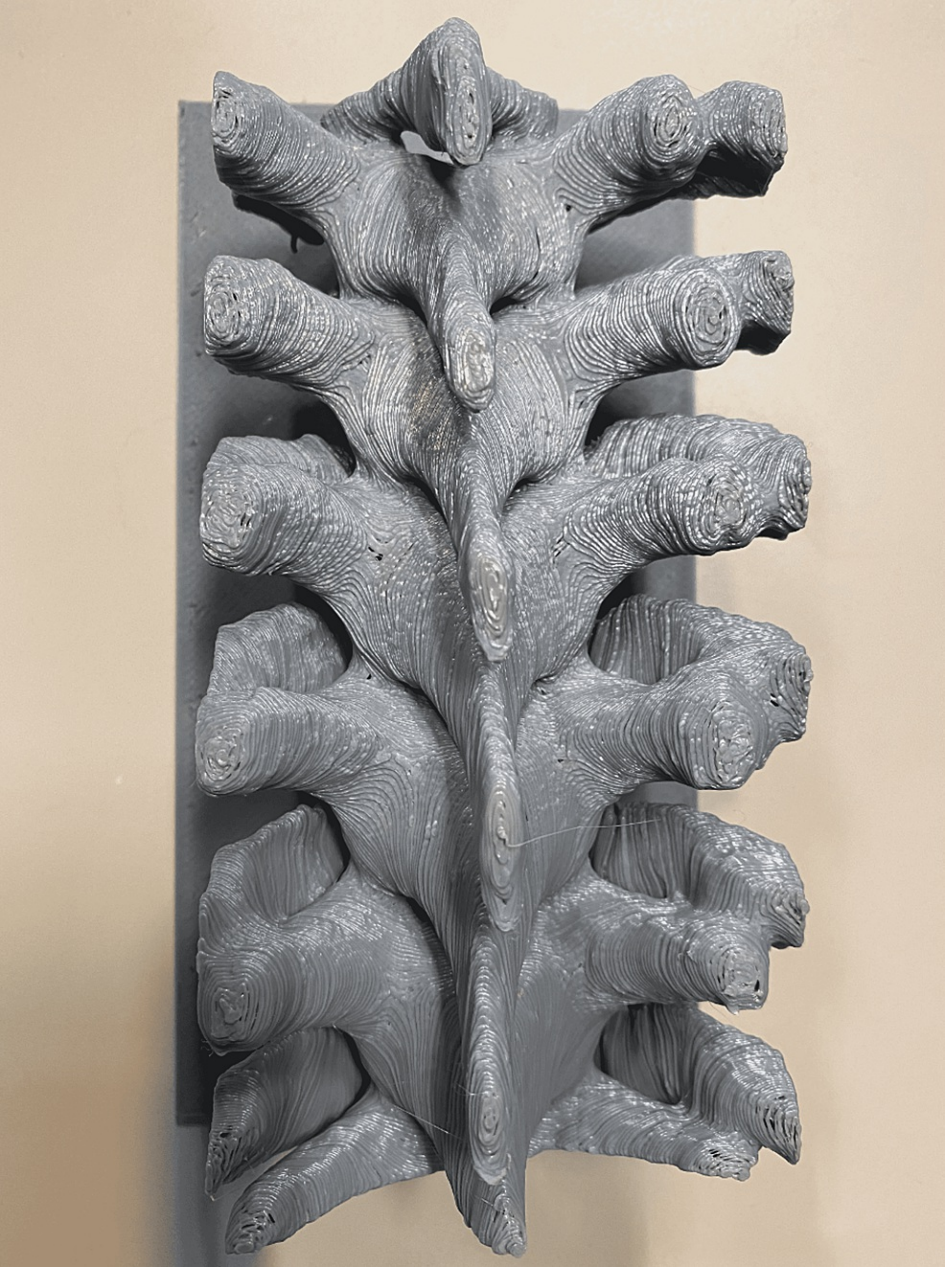
Three-dimensional-printed model of the thoracic spine, anatomically to scale.

This study was approved by Arrowhead Regional Medical Center (ARMC) Institutional Review Board (IRB) committee, protocol 22-27. Ten total resident physicians from an Accreditation Council for Graduate Medical Education (ACGME)-accredited neurosurgery residency program were recruited for this study. The 3D thoracic model was created using CT scan of a deidentified patient and spanned the thoracic four to thoracic nine levels. The model was created utilizing a free online CAD software (www.tinkercad.com) and a thermoplastic polylactic acid (PLA) filament (Figure 1). Three 3D-printed thoracic spine models were made from one roll, each of which was purchased for $22. Each print was created at 0.3mm and 15% infill. Printing took approximately six hours with 300g of material. We used a total of seven models of the thoracic spine obtained using CAD from a real patient demonstrating normal spine anatomy. In total, six thoracic levels with intact pedicles were available on the models. Screws were provided by Medtronic (Minneapolis, MN).

**Figure 2:**
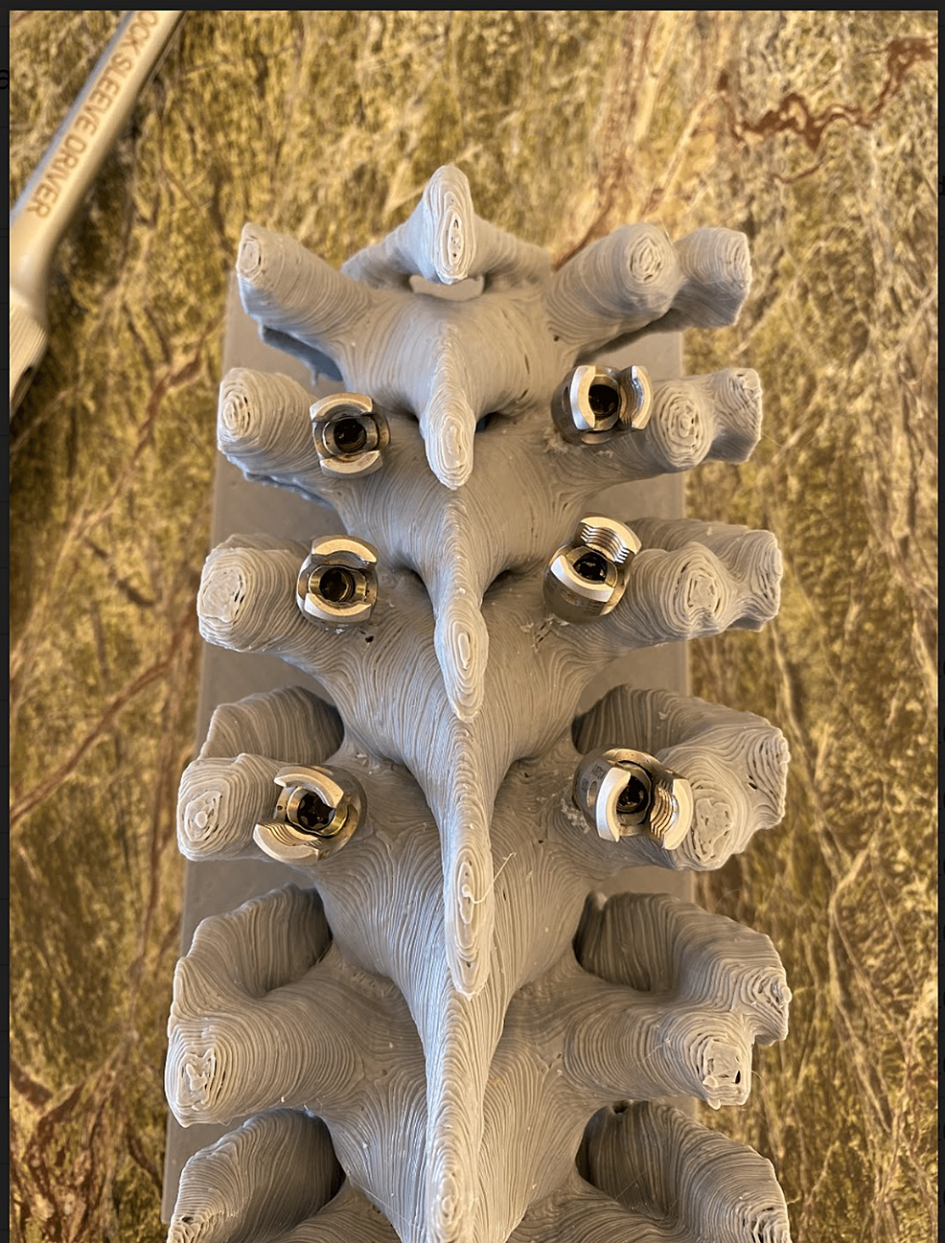
Spine model after placement of screws.

Residents were split up by academic post-graduate year and underwent identical didactic training sessions followed by a series of practical hands-on evaluation sessions. Didactic sessions consisted of a PowerPoint (Microsoft Corp., Redmond, WA) presentation that was previously recorded in video format. The training video introduced the placement of pedicle screws and reviewed the basic anatomical landmarks, specifically the transverse process and the superior articulating facet. Following the didactic sessions, residents used hand drills to create pilot holes at entry points halfway along transverse processes, 3mm inferior lateral to the superior articulating facet, which are common anatomical landmarks in the literature [4]. Bilateral screws were placed at the thoracic six and seven levels. Residents then received attending physician critiques for breaches and overall technique. The scoring was binary, whether there was a breach or not, and the critique depended on the direction of the breach. Finally, residents all placed thoracic five screws in a timed manner (Figure 2). These times and frequency of breaches are compiled in Table 1.

**Table 1:** Times and breaches for each resident trial. Min: minute; sec: second

Year	Min:sec	Medial breach	Lateral breach	Disc space breach
PGY4	0:54	x	x	x
PGY1	1:55			
PGY5	0:51			x
PGY5	0:50			
PGY6	1:29			
PGY2	1:14			

During our evaluation of these spine models as learning tools, the PGY1 and PGY2 had no breaches. Finally, the PGY6 had no breaches of thoracic five, six, and seven levels. The PGY5 had the fastest time for placement of bilateral thoracic pedicle screws at the thoracic five level.

A Likert scale was used to assess the belief in the utility of the exercise by the participants. Residents unanimously strongly agreed that this exercise was useful to demonstrate the entry point of thoracic pedicle screws. They also all strongly agreed that these models were helpful, and interest remained strong that future exercises would employ these models.

## Discussion

3D-printed models in spine surgery

Spine surgery often involves complex procedures that require considerable repetitive practice. With increasing duty hour restrictions, limited access to cadaveric specimens, and recent coronavirus disease 2019 (COVID-19) guidelines, novice trainees are now at odds with obtaining adequate practice to confidently develop basic surgical skills [5]. The use of simulation has garnered support from many neurosurgery program directors as a supplement to traditional apprenticeship learning [6]. However, despite advances in surgical simulation (i.e., virtual reality {VR} and mixed reality), very few neurosurgery residents are exposed to spine surgery simulation due to technological and budgetary constraints [7]. To improve the feasibility of surgical education, three-dimensional-printed (3DP) models are increasingly being used for surgical training and preoperative planning. These affordable models enable trainees to practice prior to operating on real patients, thereby decreasing the learning curve of surgical techniques [8,9].

Surgical training

The initial objective of using 3D-printed spine models was to improve the spatial understanding of patient anatomy. In 2015, Li et al. conducted the first randomized trial for utilizing 3DP spine models in medical education to improve the identification of spinal fractures [10]. Multiple studies that followed aimed to emulate the use of 3DP models to enhance the performance of specific procedures such as facet joint injections [10], pedicle screw instrumentation [11], spinal osteotomies [12], cervical laminectomy [13], lumbar laminectomy [14], and atlanto-occipital spinal injuries [15]. Collectively, these studies recruited medical students, neurosurgery/orthopedic residents, and attending surgeons. Study results relied mainly on quantitative metrics such as pedicle screw perforation/breach rates, in vivo canal diameter, procedure/instrumentation time, and accuracy rates. A few studies focused solely on qualitative metrics such as the Likert scale [9] and face/content validity ratings [13]. Overall, across all studies, 3DP models significantly improved performance on written and practical assessments. These results demonstrate that integration into residency programs can provide novice trainees with early exposure to challenging techniques prior to going into the operating room, thereby improving patient outcomes [15].

Preoperative planning

In addition to its use in surgical training and medical education, 3DP technology has been shown to be beneficial for experienced surgeons. Recent studies show that 3DP models can enhance patient consultations by helping patients and their families understand their anatomy and surgical plan, thereby increasing patient consent rates and compliance and decreasing patient anxiety [16]. Advances in 3DP technology have enabled the creation of inexpensive 3D-printed guides created from a patient’s preoperative CT imaging. These patient-specific guides are an excellent tool to minimize the risks of complications by helping surgeons determine accurate entry points and trajectories for pedicle screw insertion [17]. Utilizing these guides has contributed to significantly decreased preoperative preparation, operative duration, intraoperative bleeding, radiation exposure, and risk of complications in pedicle screw fixation [18-20].

Limitations

Despite the numerous benefits of 3DP models, many studies state significant limitations that hinder a realistic training experience. Many models lack relevant intraoperative features such as crucial neurovascular elements, ligamentous structures, and coagulation for hemostasis [8,10,12,13]. In addition, Park et al. [11] and other studies [21] report the unrealistic non-osseous feeling of spine models, as well as the failure to recreate dynamic spinal kinematics. Without these features, trainees may be subject to decreased educational value and false confidence [22]. Other notable limitations include the often-small sample sizes and low heterogeneity predisposing studies to selection bias and decreased external validity [9-12]. Future work should focus on multi-institutional studies employing various departments to ensure reproducibility. Lastly, a few studies highlight the importance of assessing the long-term retention of trainees and not just perioperative performance [15,23]. This is necessary to maintain appropriate teaching capabilities for subsequent trainees.

Literature review

Additionally, we performed a literature review to evaluate the current literature on spine models. Inclusion criteria consisted of peer-reviewed articles published within the last 10 years that evaluated the feasibility and utility of 3DP models for training related to spinal surgery. Exclusion criteria consisted of studies that do not evaluate the use of printed models specifically in spine surgery, studies with animal subjects, non-English studies, conference abstracts, poster presentations, or inaccessible articles. Ultimately, 11 articles were included in the final review (Table 2).

**Table 2:** Three-dimensionally-printed (3DP) models in spine surgery training. 3D: three-dimensional; NSG: neurosurgery; OPLL: ossification of the posterior longitudinal ligament; 2D: two-dimensional; N/A: not available

Reference	Sample size	Study objective	Study description	Results	Limitations	Future direction
Li et al., 2015 [10]	120 medical students	Evaluate the impact of 3DP on the identification of spinal fracture	Students were randomized into groups with or without 3DP models	Students in 3DP group had improved pleasure, effect, confidence, and identification of fracture compared to 2D-only group	Small sample size (selection bias), the lack of ligamentous and neurovascular structures	Development of printing technology that allows rapid printing and haptic capabilities of specific tissues
Liew et al., 2015 [9]	12 NSG residents	Use patient-specific 3DP spine models to improve spatial understanding of patient anatomy and improve patient consent	Patient-specific 3DP model was generated using CT imaging. In case 1, the model was used to obtain consent for posterior lumbar fixation case. In case 2, the model was assessed for its utility as an educational tool	With 3DP, residents reported (1) improved spatial understanding of the patient’s anatomy, disease, and surgical procedure; (2) enhanced assessment and management; and (3) teaching compared to using CT alone	Limited sample size decreases validity	Perform larger case series or randomized trial to assess if positive responses affect surgical outcomes
Bohl et al., 2020 [12]	3 spine surgeons	Develop a spine model that can replace cadaveric tissue in spine biomechanical research	L3-L5 vertebral bodies were 3DP for pedicle screw fixation	Lateral fluoroscopic views also demonstrated nearly perfect fidelity; one surgeon identified the minor medial breach; another surgeon identified the inferior breach after initially thinking good screw placement	Models lack relevant intraoperative features (vascular elements, nerve roots, fat, and ligament)	Biomechanical performance testing (screw insertional torque, axial pullout strength, and stiffness), soft tissue range of motion testing
Li et al., 2018 [17]	13 novice medical students	Use 3DP models to improve novice trainee confidence and proficiency in performing facet joint injections	Create a 3DP model of lumbar scoliosis and spondylosis to use in two subsequent training sessions for novice students	Second training sessions demonstrated significantly fewer needle readjustments, increased confidence, and better performance compared with the first sessions	Focusing only on CT-guided facet blocks weakens generalizability to other procedures. The use of inanimate phantom may inflate false confidence	Additional spine training models can be made from varying patient anatomy and spine levels. Involve radiology residents to facilitate anatomical teaching
Wu et al., 2018 [8]	90 medical students	Evaluate whether 3DP models can enhance teaching and learning environment of spatial bone anatomy and fractures	Students were divided into CT image only versus 3D-printed groups and 5-question test on fracture type and satisfaction survey	Scores on both tests were lower, and test-taking times were higher in CT image group compared to 3DP model group	Models lack soft tissue structures (nerves, vessels, and muscles); 3D printing process varied; small sample size	Reduce time requirement to process 3D models by further development of 3DP technology
Park et al., 2018 [11]	2 residents	Use real-size 3DP spine models to evaluate improvement of (1) screw instrumentation accuracy and (2) length of procedure	Two novice surgeons were instructed by seniors before placing 10 pedicle screws in patient lumbar models	37/200 screws (18.5%) perforated the pedicle cortex with a mean of 1.7mm; the latter half of the models had (1) significantly less violation and (2) less mean length of time to complete pedicle screw instrumentations than the former models	Unrealistic feeling of non-osseous 3DP models	Reduce large initial investment required for in-office production of 3D models; evaluate new materials to mimic the osseous feel of real pedicles
Bohl et al., 2020 [12]	6 medical students, 2 NSG residents	Evaluate the use of 3DP spine models to improve trainee’s knowledge and performance of spinal osteotomies	Participants were separated into written material group and 3DP model group before undergoing written and practical examinations	The 3DP model group performed significantly better on both the written and practical assessments	Small sample size, possible confirmation bias from unblinded study personnel	Use 3DP models of various spine segments to learn complex surgical and pathological concepts
Weiss et al., 2020 [13]	7 residents, 5 attendings	Develop a cervical spine laminectomy simulator capable of measuring operative performance and assessing face, content, and construct validity	Controlled trial using 3DP models assessed performance (intrathecal pressure, complication rate, and blood loss), face, and content validity	Mean face and content validity ratings were 4/5; significant difference in intrathecal pressure, procedure time, and complication rate between experts and novices	The lack of coagulation and hemostasis confounded blood loss and face validity	Incorporate coagulable blood into multi-institutional study that tests varying levels between novice and experts
Clifton et al., 2020 [24]	4 NSG residents, 3 nurse practitioners, 3 physician assistants	Investigate 3DP techniques to create dynamic educational models that demonstrate kinematic and physiologic concepts	3DP dynamic versus static models underwent flexion and extension under fluoroscopy to compare educational benefit of physiologic concepts	The flexible 3D-printed model more accurately reflected in vivo measurements of canal diameter changes during dynamic positioning; flexible models were more successful in teaching the physiologic concepts of spinal canal changes during flexion and extension than the static 3D-printed model	The absence of a simulated discoligamentous complex fails to recreate realistic cervical kinematics; focus on immediate experiential learning without long-term concept retention validation; models were based on OPLL pathology without healthy control 3D models	Utilize dynamic 3D-printed models to simulate additional biomechanical concepts in pathological and normal controls
Chainey et al., 2021 [14]	4 expert NSG attendings, 3 residents	Use 3DP models to compare performance of lumbar laminectomy between resident and expert neurosurgeons	Analyze video and eye tracking during a drilling task to evaluate differences in hand-eye coordination and fine movement control	Residents had more jumping events, greater jump distances, and longer post-jump fixation durations when compared to expert neurosurgeons	Small sample size limited to a single institution’s neurosurgery division; drilling task was less complicated and lacked surrounding soft tissue structures seen in realistic procedures	Display expert’s hand-eye coordination features to teach novices to more accurately guide fine control movements
Öztürk et al., 2022 [15]	N/A	Use 3DP models to evaluate improvement of surgery duration, radiation exposure, blood loss, and the accuracy of pedicular screw placement for atlanto-occipital spinal C-type injuries	Residents were briefed before performing a pedicle screw implantation procedure. They were then assessed by a senior	Statistically significant decrease in instrumentation time, blood loss, medial axis encroachment, and intraoperative fluoroscopy numbers in the 3D model-assisted surgery group compared to the conventional surgery group	Randomized non-controlled study; assessed only perioperative parameters and not long-term clinical outcomes	Perform randomized controlled trials in other fields within more varied clinical contexts

Future directions

Given these limitations, many ongoing studies are focused on recapitulating the intraoperative features to maximize the educational value of 3DP models. Companies such as ImmersiveTouch (Chicago, IL) have developed VR platforms with haptic feedback for pedicle fixation [23]. Similarly, recent pilot studies have tested dynamic 3D models that incorporate neurovascular and soft tissue structures to recreate the biomechanics and kinematics of the spine [25]. Other groups are developing non-biohazardous replica blood that coagulates, in addition to pressure sensors that measure nerve root compression, traction, and dural tension [23,26]. Pressure sensors have also been used to provide instant feedback (via buzzer/light) to indicate suprathreshold maneuvers. This aims to speed up the trainee’s learning curve to reliably perform safer maneuvers on live patients [13]. Other initiatives are striving to improve the access and feasibility of 3DP technology. The SpineBox, developed by Anatomics (Melbourne, Australia), represents the first open-access simulator that aims to provide institutions across the globe with a downloadable and economical means of surgical simulation [27-31]. Ultimately, 3DP technology in spinal surgery is still in its infancy. Future studies must be carried out to find innovative ways to provide reliable yet feasible surgical training.

## Conclusions

We sought to evaluate the feasibility of creating a cheap, practical, easy-to-produce model for the training of neurosurgical residents. With the advent of improved intraoperative imaging modalities, freehand techniques for pedicle screw placement may become a skill that is not as prominent as it once was. Accordingly, residents would benefit from models that may test entry point, trajectory, and a lack of breaching when placing pedicle screws. The small and mobile nature of each model allows for the individual to check for breaches by looking down the central canal, out laterally, and into the disc space. The thoracic spine provides some of the most technically difficult anatomies to operate on in the spine. The tight central canal and proximity to the lungs add to this complexity. Several protocols exist online for the creation of 3D-printed spine models. The implementation of spine models into the armamentarium of training tools available to residents is now inexpensive and easily obtainable. We demonstrate the teaching of thoracic pedicle screw placement via the use of 3D models. Such models are affordable and easy to use and have received positive feedback from neurosurgery residents in the use of demonstrating proper technique and placement of thoracic pedicle screws.

## Appendices

Likert scale questionnaire

1. The utilization of 3D-printed models for the purposes of learning screw placement is useful.

Strongly Disagree

Disagree

Neutral

Agree

Strongly Agree

2. I have benefited from this exercise.

Strongly Disagree

Disagree

Neutral

Agree

Strongly Agree

3. More simulations involving 3D-printed models would be helpful for my training.

Strongly Disagree

Disagree

Neutral

Agree

Strongly Agree

4. 3D-printed models provide a low-cost, easily obtainable training tool.

Strongly Disagree

Disagree

Neutral

Agree

Strongly Agree
